# A Dual-Drug Nanocarrier Strategy for Ocular Fungal Infections: Micelles Embedded in Electrospun Nanofibers

**DOI:** 10.3390/molecules31081235

**Published:** 2026-04-08

**Authors:** Egemen Uzel, Meltem Ezgi Durgun, Neriman Aydilek, Mayram Hacıoğlu, Sevgi Güngör, Yıldız Özsoy

**Affiliations:** 1 Department of Pharmaceutical Technology, Faculty of Pharmacy, Istanbul University, Istanbul 34126, Türkiye; egemenuzel@gmail.com (E.U.); neriman.aydilek@ogr.iu.edu.tr (N.A.); sevgi.gungor@istanbul.edu.tr (S.G.); 2 Department of Pharmaceutical Technology, Faculty of Pharmacy, Istanbul Health and Technology University, Istanbul 34275, Türkiye; meltem.durgun@istun.edu.tr; 3 Department of Pharmaceutical Microbiology, Faculty of Pharmacy, Istanbul University, Istanbul 34126, Türkiye; mayramtuysuz@gmail.com

**Keywords:** ocular drug delivery, electrospinning, nanofiber inserts, polymeric micelles, posaconazole, dexketoprofen trometamol

## Abstract

Ocular fungal diseases are associated with severe infection and pain and, in advanced stages, can lead to vision loss. Current treatment options are limited to the topical application of conventional drugs, and the bioavailability of these drugs is quite limited due to ocular barriers. In this study, a dual-drug nanodelivery system was developed to improve intraocular drug delivery by combining antifungal and anti-inflammatory therapies. Posaconazole (PSC), a broad-spectrum triazole antifungal agent, and dexketoprofen trometamol (DKP), a rapidly acting nonsteroidal anti-inflammatory drug, were co-loaded onto polymeric micelles and then incorporated into electrospun poly(vinyl alcohol)/poly(vinylpyrrolidone) (PVA/PVP) nanofiber intraocular implants. DSC, XRD, FTIR, and FESEM analyses showed that both APIs were successfully converted into nanofiber form without disrupting the micelle structure. Comparative studies with DKP solution and PSC commercial oral suspension (Noxafil^®^ 40 mg/mL) showed that the produced micelle-loaded nanofibers provided sustained release and significantly increased ex vivo ocular permeation and penetration. In vitro antifungal activity tests demonstrated efficacy against *Candida albicans*, and HET-CAM toxicity tests showed that the micelle-loaded nanofibers were non-irritating and suitable for ocular application. Overall, the micelle-loaded electrospun nanofiber ocular inserts developed in this study represent a promising platform for combined antifungal and anti-inflammatory ocular therapy.

## 1. Introduction

Fungal ocular diseases are less common than bacterial or viral infections; however, if not diagnosed and treated promptly, they can lead to severe morbidity, including vision loss and even blindness, and are often accompanied by pain and inflammation. Their incidence is particularly high in tropical and subtropical regions. Globally, it is estimated that more than one million cases of fungal keratitis occur each year, with serious outcomes—including enucleation—reported in approximately 8–11% of cases. The burden of these infections is also increasing in temperate regions, driven by factors such as the rising use of contact lenses and a higher rate of environmentally related ocular trauma [1,2].

Topical antifungal agents are generally the first-line therapy for these conditions. In severe cases, treatment is often escalated to include systemic therapy and/or combination regimens in addition to topical administration. Systemic antifungals may be used in the management of fungal ocular diseases, and combination approaches frequently incorporate steroidal or non-steroidal anti-inflammatory drugs to alleviate inflammation and pain [3]. Nevertheless, both systemically administered and conventional topical formulations typically exhibit low ocular bioavailability due to the complex barrier properties of ocular tissues, including the tear film, cornea, conjunctiva, sclera, blood–aqueous humor barrier (iris–ciliary body), lens, and blood–retina barrier, which collectively present both lipophilic and hydrophilic obstacles to drug permeation and retention. For example, the ocular bioavailability of conventional eye drops is generally less than 5% [4,5].

To overcome these limitations, research interest in controlled-release platforms and nanocarrier-based delivery systems has increased substantially. Such approaches aim to prolong precorneal residence time, enhance tissue penetration, and improve ocular bioavailability, thereby increasing therapeutic efficacy while minimizing adverse effects [6,7]. In addition, accumulating evidence suggests that combined dosage forms can further improve therapeutic outcomes in various ocular disorders [8,9].

Micelles, one of the nanocarrier systems developed for ocular delivery, are nanoscale structures typically composed of a hydrophobic core and a hydrophilic shell formed from amphiphilic copolymers or surfactants. They are colloidal drug delivery systems that spontaneously assemble in solution when the polymer/surfactant concentration exceeds the critical micelle concentration (CMC) [10]. Amphiphilic surfactants or diblock copolymers self-associate to form micellar structures once a threshold concentration and/or temperature is reached [7]. Micelles generally range from 5 to 200 nm in size and may adopt diverse morphologies, including spherical, cylindrical, or star-like structures. A key feature distinguishing micelles from many other carrier systems is their ability to encapsulate not only lipophilic drugs but also hydrophilic compounds, which makes them well suited for combination drug therapy. Moreover, micellar systems can enhance the corneal permeability of topically administered drugs and are therefore considered promising candidates for delivering therapeutics targeting ocular tissues [11,12,13].

Nanofiber-based ocular inserts represent another drug delivery platform that has attracted increasing research interest for ocular applications. Nanofibers are nanoscale solid fibrous structures that can be fabricated from natural or synthetic polymers and typically exhibit diameters in the range of 1–1000 nm. Owing to this distinctive architecture, nanofibers are considered promising drug delivery systems and can be engineered to achieve different drug-release kinetics. As a delivery platform, nanofibers offer several advantages, including their suitability for the controlled release of both hydrophilic and lipophilic active substances as well as biomolecules (e.g., antibiotics and proteins) [14]. Additional benefits include a high surface area-to-volume ratio, high porosity, and a structure that can resemble the extracellular matrix of tissues. Moreover, their production methods are often simpler and less costly than those of many other nanostructured delivery systems. Among the available fabrication approaches, electrospinning is the most widely used technique for producing nanofibers [15].

Electrospinning generates ultrafine fibers by applying a high-voltage, low-current electrical potential and is a simple and robust method for producing nanofibers with diameters typically in the 10–1000 nm range [16]. One of its principal advantages is the high degree of control it provides over fiber morphology, enabling researchers to tailor nanofiber properties for specific applications such as tissue engineering, drug delivery, and filtration. In addition, electrospinning is a versatile technique compatible with a broad range of polymers, including natural polymers (e.g., hyaluronic acid, chitosan, dextran, gelatin, and collagen), synthetic polymers (e.g., poly(lactic acid), poly(lactic-co-glycolic acid), poly(ε-caprolactone), poly(vinyl alcohol), and poly(vinylpyrrolidone), and their blends. This versatility facilitates the production of nanofibers with diverse physicochemical characteristics. In addition, its ability to be used in combination with other drug delivery systems (nanoparticles, dendrimers, micelles, etc.) provides a significant advantage [17,18,19].

Posaconazole (PSC) is a broad-spectrum second generation triazole antifungal that exhibits low minimum inhibitory concentration (MIC) values against a wide range of fungal strains. It exerts its antifungal activity by altering the fungal cell membrane, primarily through inhibition of ergosterol biosynthesis via interaction with lanosterol 14α-demethylase (CYP51). PSC is classified as a Biopharmaceutics Classification System (BCS) Class II compound, characterized by low aqueous solubility and high permeability. It is highly lipophilic, with a logP of 5.5 [20,21,22]. Currently, there is no commercially available ocular formulation of PSC. Notably, case reports have described therapeutic benefit even when PSC oral commercial suspension (Noxafil^®^ 40 mg/mL) was diluted and administered topically to the eye in resistant, fungus-derived ocular infections unresponsive to other antifungal treatments [23,24]. Recent studies have increasingly explored the potential of posaconazole for ocular antifungal therapy. Durgun and colleagues optimized micellar formulations that enhanced the aqueous solubility of PSC and improved its permeability across ocular tissues [25]. In subsequent work, they focused on micelle-based in situ gelling systems to further increase the ocular bioavailability of PSC, reporting high antifungal activity without inducing ocular toxicity [26].

Nonsteroidal anti-inflammatory drugs (NSAIDs) are preferred in ocular inflammation to alleviate inflammation, pain, and photophobia. They play a critical supportive role in the treatment of ocular infections, particularly in ocular fungal infections, as an alternative to corticosteroids, which tend to exacerbate the infection [27]. Dexketoprofen trometamol (DKP) is the active S-(+)-enantiomer of ketoprofen. It has a high onset of analgesic effect and, due to its trometamol salt form, has very high sucrose solubility. It offers a better bioavailability profile and higher tolerability compared to ketoprofen. It acts by inhibiting both cyclooxygenase-1 (COX-1) and cyclooxygenase-2 (COX-2), thereby suppressing the synthesis of prostaglandins, which are key mediators of inflammation and pain [28]. Although studies on ocular drug delivery systems for various NSAIDs such as diclofenac, ketorolac, and nepafenac exist in the literature, DKP has not yet been investigated for ocular application [29]. Considering its high solubility (BCS Class I) and rapid anti-inflammatory activity [30,31], the aim is to alleviate inflammation-related tissue damage by adding DKP to the current formulation and to synergistically complement PSC in antifungal therapy.

In this study, we aimed to design an innovative “micellar nanofiber” system to enhance the aqueous solubility of PSC and improve the penetration of both active agents (PSC and DKP) into ocular tissues. To this end, drug-loaded micelles were first prepared and subsequently incorporated into polymeric nanofibers via electrospinning to produce solid-state ocular inserts. The developed combined nanocarrier platform was systematically evaluated in terms of physicochemical characterization, in vitro drug release, ex vivo permeation and penetration, and antifungal efficacy, with the goal of contributing to the existing literature on advanced ocular drug delivery systems.

A review of the literature reveals no studies describing the integration of a dual-drug antifungal-anti-inflammatory micellar electrospun nanofiber ocular implant. This study is the first of its kind. While polymeric micelles and electrospun nanofibers have been studied separately for intraocular drug delivery, the combination of “micellar nanofibers” for the simultaneous delivery of PSC, an antifungal agent, and DKP, a nonsteroidal anti-inflammatory drug, has not been previously reported. This combined delivery system strategy is designed to overcome the different biopharmaceutical limitations of each API by increasing the water solubility of PSC, controlling the release kinetics of both DKP and PSC, and improving ocular permeability-on-penetration, thus offering a multifunctional approach for the management of fungal eye infections accompanied by inflammation and pain.

## 2. Results

### 2.1. Preparation and Characterization of PSC/DKP-Loaded Polymeric Micelles

PSC/DKP-loaded polymeric micelles were successfully obtained with nanoscale dimensions appropriate for ocular delivery. Dynamic light scattering measurements showed a mean hydrodynamic diameter of 11.01 ± 0.06 nm with a low PDI of 0.078, indicating a narrow size distribution. The zeta potential of the optimized micellar formulation was −1.68 ± 0.23 mV. Drug incorporation into the micellar system was high for both actives. Encapsulation efficiencies were 97.55 ± 4.10% for DKP and 98.67 ± 4.71% for PSC. All results are summarized in Table 1.

### 2.2. Characterization of Spinning Solutions

**Figure 1:**
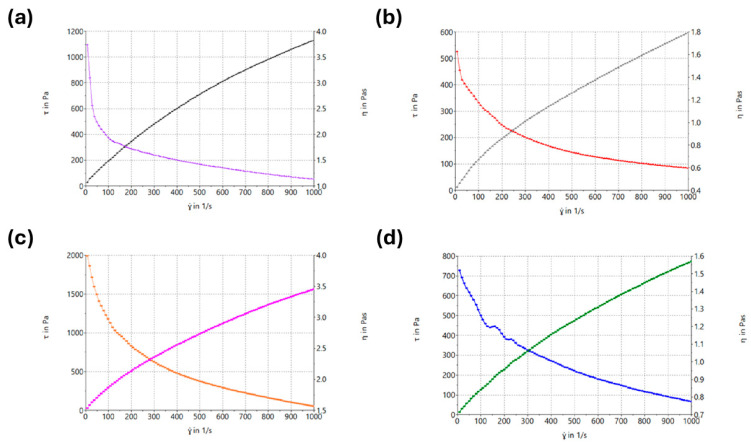
Rheograms of electrospinning solutions of nanofiber formulations. (**a**) Nanofiber-A electrospinning solution, (**b**) Nanofiber-P electrospinning solution, (**c**) Nanofiber-AP electrospinning solution, and (**d**) Nanofiber-P-(−M) electrospinning solution.

Spinning solution properties strongly govern jet stability and fiber morphology. Thus, electrical conductivity and rheological behavior were assessed as key predictors of electrospinnability. Rheograms of the electrospinning solutions are shown in Figure 1. Rheological data were fitted to mathematical models and compared based on correlation coefficients (r). The best agreement was obtained with the Herschel–Bulkley model for all tested solutions. Rheology and conductivity data are given in Table 2.

### 2.3. Characterization of Micellar Nanofiber Inserts

#### 2.3.1. Drug Content and Encapsulation Efficiency

The drug content in the nanofiber inserts was measured by HPLC using the sample preparation procedure described in “Section 4.2” of this article. Encapsulation efficiencies determined from samples collected at different locations of the nanofiber mats are presented in Table 3.

#### 2.3.2. Thickness, Diameter, and Weight

Physical characterization of micelle-loaded nanofiber inserts showed in Table 3.

#### 2.3.3. FESEM Imagining and Fiber Diameter

**Figure 2:**
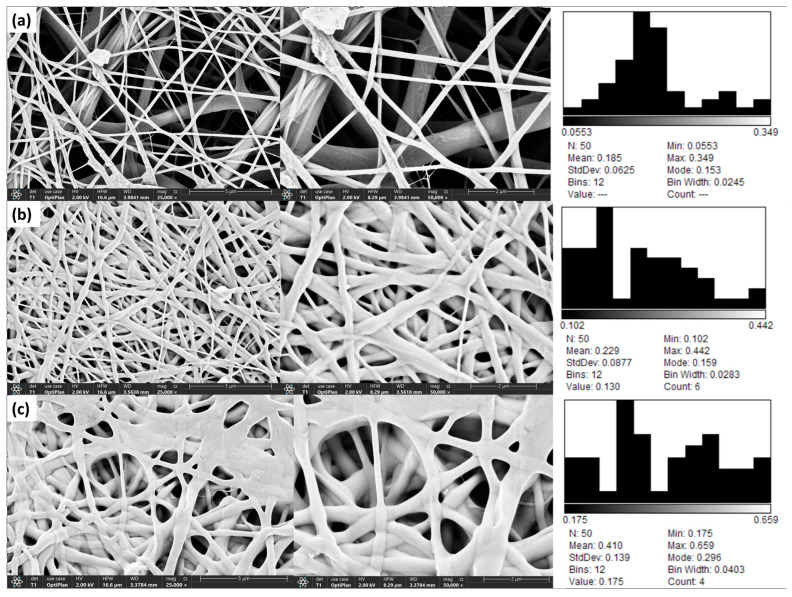
FESEM imaging findings at 25,000× and 50,000× magnification (*n* = 50): (**a**) Nanofiber-A, (**b**) Nanofiber-P, and (**c**) Nanofiber-AP.

FESEM images and fiber diameter distribution histograms are provided in Figure 2.

#### 2.3.4. Differential Scanning Calorimetry (DSC) Analysis

**Figure 3:**
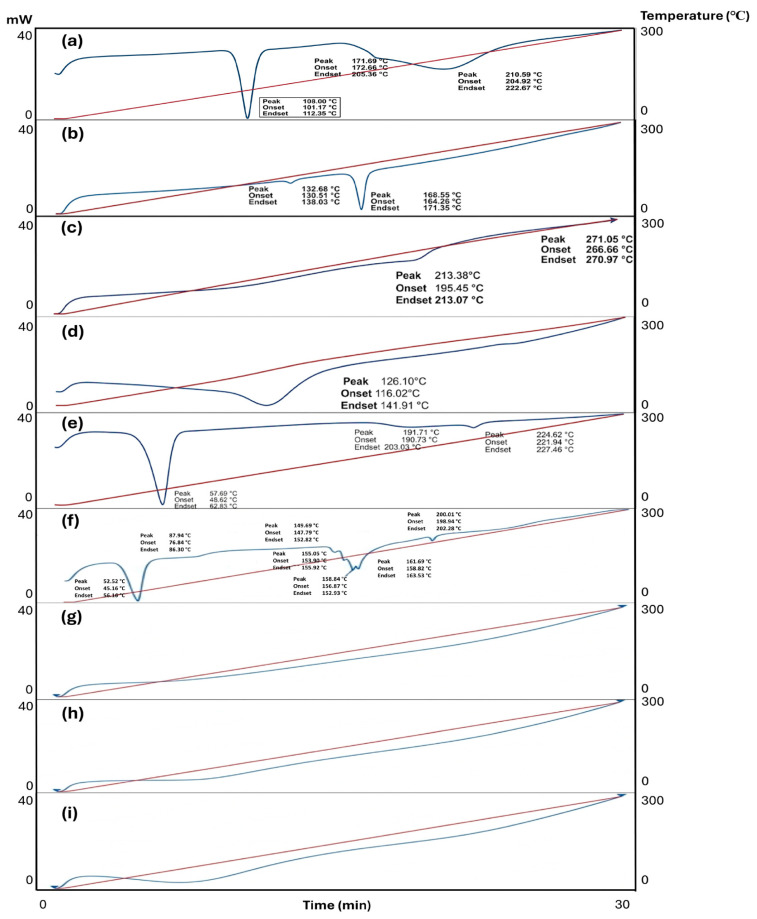
Thermograms of the DSC analysis. (**a**) DKP, (**b**) PSC, (**c**) PVA, (**d**) PVP, (**e**) PLU, (**f**) MIX, (**g**) NF-A, (**h**) NF-P and (**i**) NF-AP. DSC: Differential scanning calorimetry; PSC: Posaconazole; DKP: Dexketoprofen trometamol; PVA: poly(vinyl alcohol); PVP: poly(vinylpyrrolidone); PLU: Pluronic^®^ F; NF-A: Nanofiber-A; NF-P: Nanofiber-P; NF-AP: Nanofiber-AP.

DSC thermograms showed in Figure 3.

#### 2.3.5. X-Ray Diffraction (XRD) Mapping

**Figure 4:**
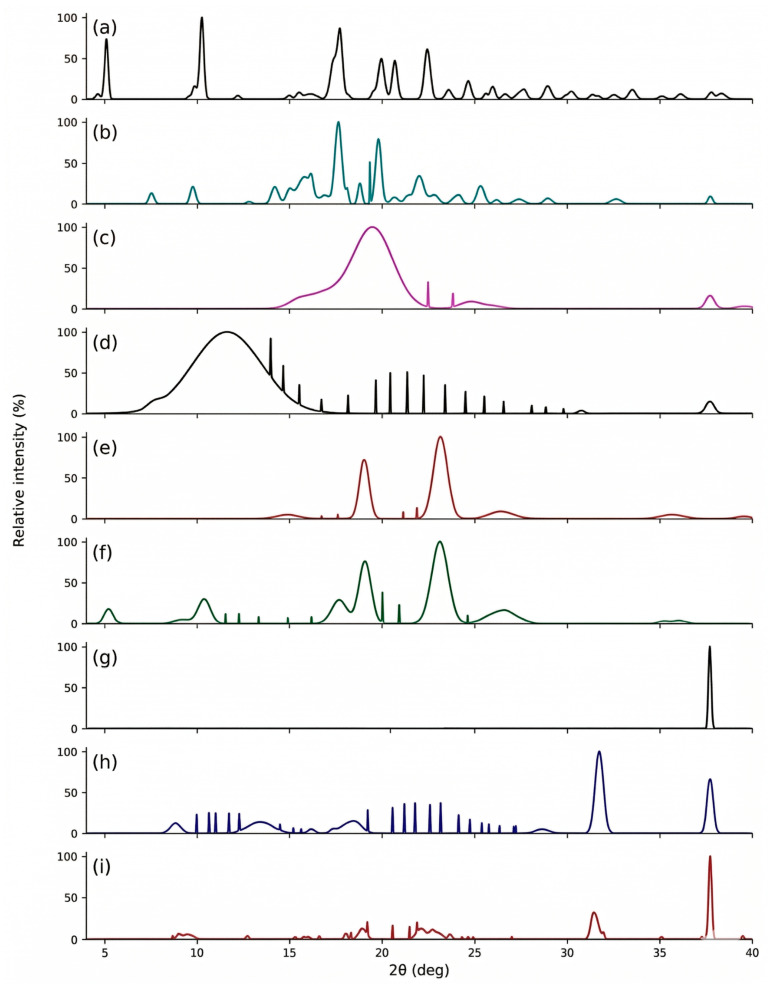
Results graphs of XRD analysis. (**a**) DKP, (**b**) PSC, (**c**) PVA, (**d**) PVP, (**e**) PLU, (**f**) MIX, (**g**) NF-A, (**h**) NF-P and (**i**) NF-AP. XRD: X-ray Diffraction; PSC: Posaconazole; DKP: Dexketoprofen trometamol; PVA: poly (vinyl alcohol); PVP: poly(vinylpyrrolidone); PLU: Pluronic^®^ F; NF-A: Nanofiber-A; NF-P: Nanofiber-P; NF-AP: Nanofiber-AP.

XRD diffractograms presented in Figure 4.

#### 2.3.6. Fourier-Transform Infrared Spectroscopy (FTIR, ATR-FTIR) Analysis

**Figure 5:**
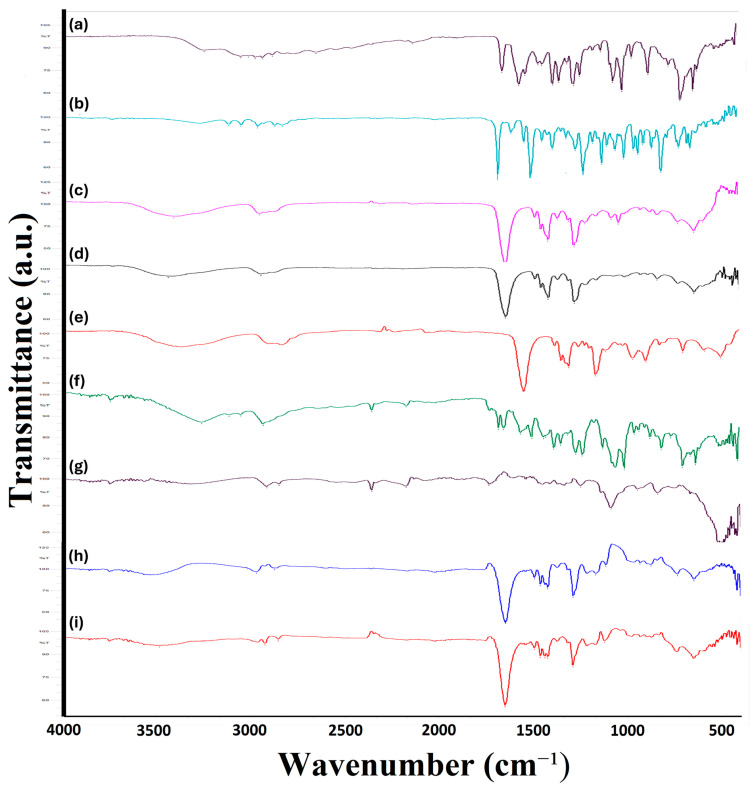
Results graphs of ATR-FTIR analysis (**a**) DKP, (**b**) PSC, (**c**) PVA, (**d**) PVP, (**e**) PLU, (**f**) MIX, (**g**) NF-A, (**h**) NF-P and (**i**) NF-AP. FTIR: Fourier Trans-form Infrared; PSC: Posaconazole; DKP: Dexketoprofen trometamol; PVA: poly(vinyl alcohol); PVP: poly(vinylpyrrolidone); PLU: Pluronic^®^ F; NF-A: Nanofiber-A; NF-P: Nanofiber-P; NF-AP: Nanofiber-AP.

ATR-FTIR spectra of the pure components, physical mixtures, and produced nanofibers are presented in Figure 5.

### 2.4. In Vitro Drug Release

**Figure 6:**
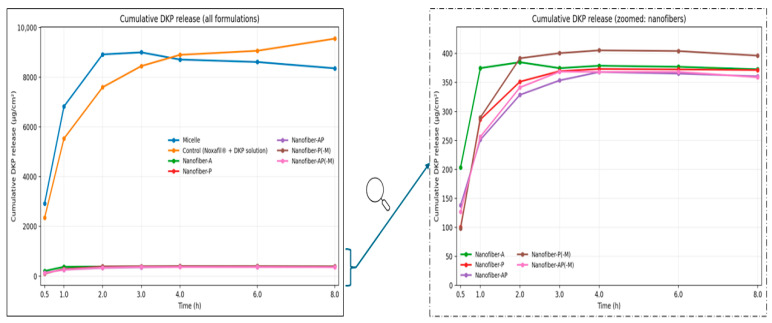
Cumulative DKP release profiles for micelle, nanofibers and control (*n* = 3). Nanofiber curves are not clearly visible due to overlapping at low concentrations; therefore, a zoomed-in plot is provided on the right, as indicated by the arrow.

**Figure 7:**
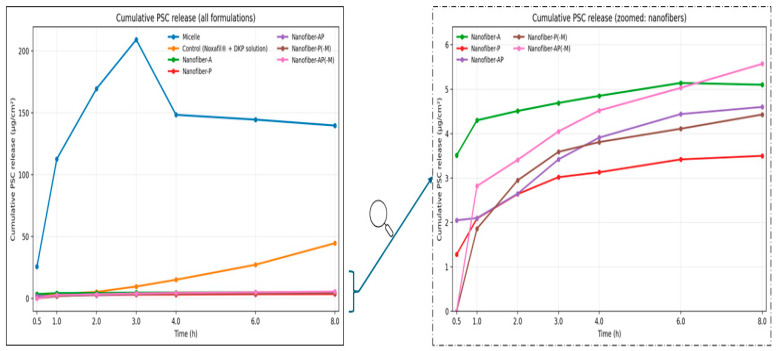
Cumulative PSC release profiles for micelle, nanofibers and control (*n =* 3). Nanofiber curves are not clearly visible due to overlapping at low concentrations; therefore, a zoomed-in plot is provided on the right, as indicated by the arrow.

In vitro release was evaluated for the micellar formulation, a Noxafil^®^ + DKP control solution diluted to the same DKP/PSC concentrations as the micelles, and the nanofiber inserts Nanofiber-A, -P, -AP, -P(−M), and -AP(−M) using a 0.45 µm cellulose acetate membrane in Franz diffusion cells. Here, the suffix “(−M)” denotes nanofiber formulations prepared without micellar encapsulation (i.e., PSC and DKP were incorporated directly into the polymer solution rather than being pre-loaded into micelles). Cumulative release (µg/cm^2^) was monitored over 8 h. Time-dependent drug release graphs from DKP and PSC carrier systems are given in Figure 6 and Figure 7, respectively.

### 2.5. Ex Vivo Permeation and Penetration Studies

#### 2.5.1. Ex Vivo Permeation Studies

**Figure 8:**
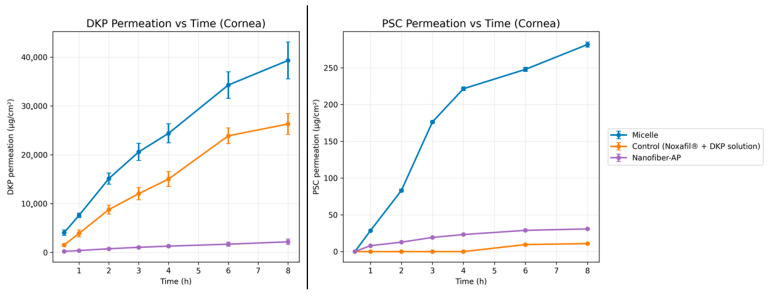
Ex vivo cumulative corneal DKP and PSC permeation profiles of Micelles, Nanofiber-AP and control (Noxafil^®^ + DKP solution) (*n* = 3).

**Figure 9:**
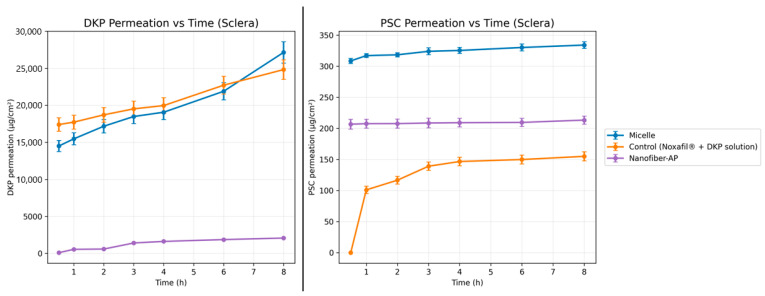
Ex vivo cumulative scleral DKP and PSC permeation profiles of Micelles, Nanofiber-AP and control (Noxafil^®^ + DKP solution) (*n* = 3).

Ex vivo permeation experiments were conducted using bovine cornea and sclera for the micellar formulation, Nanofiber-AP, and a control solution consisting of a mixture of Noxafil^®^ diluted to 250 µg/mL PSC and DKP solution at 10 mg/mL. The studies were performed using Valia–Chien diffusion cells as described before. The donor and receptor phase volumes were 2 mL for corneal cells and 3.4 mL for scleral cells. The diffusion area was 20.7 mm^2^ for corneal cells and 7.2 mm^2^ for scleral cells. Following isolation and cleaning, corneal and scleral tissues were mounted in the diffusion cells, and the receptor compartment was filled with artificial tear fluid containing 0.1% (*w*/*v*) SLS. Cumulative permeated amounts of DKP and PSC were calculated as µg/cm^2^ over 8 h. Corneal and scleral permeation results for both DKP and PSC are shown in Figure 8 and Figure 9, respectively. Following permeation testing, permeation parameters were calculated based on the ex vivo permeation data, and the calculated parameters are presented in Table 4.

#### 2.5.2. Ex Vivo Penetration Studies

**Figure 10:**
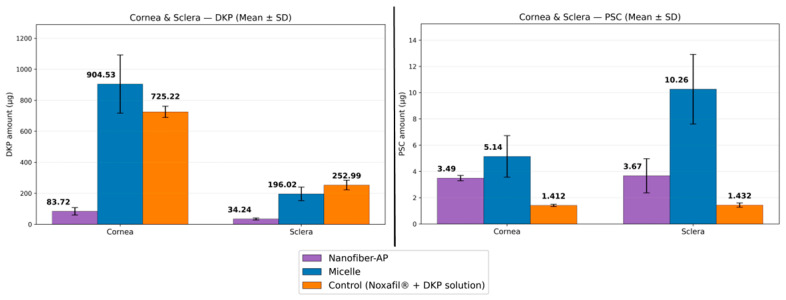
Ex vivo cumulative ocular DKP and PSC penetration profiles of Micelle, Nanofiber-AP and Control (Noxafil^®^ + DKP solution) (*n* = 3).

Corneal and scleral penetration results for both DKP and PSC are shown in Figure 10.

### 2.6. Antifungal Activity (Disk Diffusion)

Inhibition zone diameters (mm) obtained from the disk diffusion assay for the placebo, micellar formulation, Nanofiber-AP, the Noxafil^®^ + DKP mixture at the same active concentrations as the micellar formulation, and the Noxafil^®^ + DKP mixture at the same active concentrations as Nanofiber-AP are presented in Table 5.

### 2.7. HET-CAM Irritation Test

**Figure 11:**
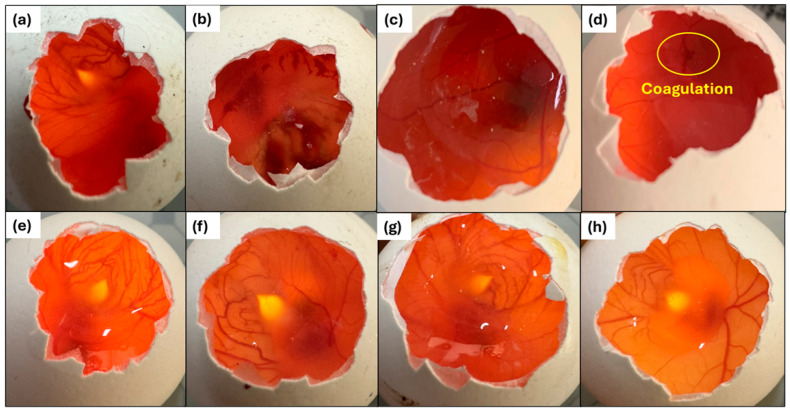
HET-CAM: Representative result images for prepared formulations (Nanofiber-AP, Micelle), Placebos and controls. (**a**) Negative control, (**b**) Positive control, (**c**) Placebo, (**d**) PSC solution (Noxafil), (**e**) DKP solution, (**f**) Mixture of Noxafil and DKP solution, (**g**) Micelles, (**h**) Nanofiber-AP.

The HET-CAM irritation assessment was performed on White Leghorn fertilized hen’s eggs as described in Section 4.10. Representative images obtained from the assay are shown in Figure 11, while the irritation scores of the formulations and controls are provided in Table 6. The corresponding classification categories for HET-CAM scores are presented in Section 4.10. Each sample was applied to three different eggs (*n* = 3), and all CAM membranes were monitored for 300 s.

During evaluation, coagulation within the first 30 s was observed in only one egg treated with the diluted Noxafil^®^ suspension (Figure 11). According to the time-dependent HET-CAM scoring scheme, this finding corresponds to a score of 9. As the experiment was conducted in triplicate (*n* = 3), this score was divided by three to obtain the mean irritation score, which was subsequently categorized as “slight” irritation based on the HET-CAM classification scheme. In contrast, no observable alterations in the vascularized chorioallantoic membrane were detected for any of the other actives or formulated systems.

## 3. Discussion

In fungal ocular diseases, infection alone does not cause tissue damage. The process, accompanied by inflammation, progresses very painfully and the healing process is delayed [2]. The two APIs included in the formulation in this study were actually selected based on this consideration. PSC disrupts the structural integrity and fluidity of the fungal cell membrane and increases membrane permeability by inhibiting lanosterol 14α-demethylase (CYP51), a key enzyme involved in ergosterol biosynthesis. As a result, it leads to the death of the fungal cell and exhibits an anti-infective effect [22]. On the other hand, DKP, an NSAID, inhibits cyclooxygenase (COX-1 and COX-2) enzymes and provides an additional therapeutic benefit by reducing prostaglandin-mediated inflammatory responses [28]. The combined delivery of PSC and DKP in a single nanocarrier system offers a synergistic therapeutic strategy by simultaneously targeting the infectious agent and associated inflammatory processes, which can increase the overall treatment efficacy in fungal eye diseases.

### 3.1. Preperation and Characterization of PSC/DKP-Loaded Polymeric Micelles

The micelles produced were found to be ultra-small (11.01 ± 0.06 nm, seen in Table 1) in terms of particle size. Such ultrasmall and relatively monodisperse systems are advantageous for ocular applications, as they can spread rapidly within the tear film and may facilitate close contact with the corneal surface, potentially supporting drug transport toward anterior ocular tissues. The advantage of this ultra-small size becomes even more prominent, especially when considering the pore openings of ocular tissues. It is known that for successful corneal permeation, the particle size of nanocarriers should be smaller than 100 nm [32]. In particular, nanocarriers smaller than 20 nm can easily pass through the spaces between the collagen fibers of the corneal stroma [33].

The zeta potential of the micelles was found to be within the neutral range of mV (seen in Table 1). This near-neutral surface charge is consistent with sterically stabilized micellar systems, where colloidal stability is primarily provided by the hydrated polymeric corona rather than strong electrostatic repulsion. For nanocarriers to be more stable, the zeta potential is expected to be in the range of ±30 mV [34]. However, considering that the mucin layer on the ocular surface is negatively charged, it is known that highly charged colloids, especially positively charged ones, can interact strongly with the ocular mucosa and epithelial surfaces. On the other hand, neutral systems can easily pass through mucin [35].

Overall, these findings are consistent with previous reports in the literature. The micelle characteristics observed in the present study were in agreement with the results reported by Durgun et al. [25]. In addition, similar particle size and zeta potential values have been obtained in the micellar formulation study by Suksiriworapong et al., who prepared itraconazole-loaded micelles using TPGS [36], supporting the comparability of the present micellar system with previously reported TPGS-based micellar platforms.

Encapsulation activity was demonstrated, showing that both APIs could be loaded into the micelle structure with high efficiency (seen in Table 1). For PSC—an antifungal with low aqueous solubility—high loading within nanoscale micelles is particularly relevant, as it supports enhanced apparent solubility and enables delivery in an aqueous ocular-compatible form. Although DKP is intrinsically more soluble, its co-incorporation at high efficiency supports the feasibility of a combined therapy approach within a single nanocarrier system.

### 3.2. Characterization of Spinning Solutions

Electrospinning solutions were developed to enable the formation of continuous nanofibers and to allow incorporation of PSC and DKP either directly or via the micellar dispersion. In addition to the micelle-loaded systems, a PVP-based solution containing PSC and DKP at the same concentrations but without micellar encapsulation (i.e., the non-micellar counterpart of the Nanofiber-P system, denoted as P(−M)) was also evaluated in order to specifically assess the influence of micelles on the flow behavior and processability of the polymeric spinning solution. Conductivity values were within a range compatible with stable electrospinning.

In rheology studies, all solutions exhibited n values below 1 (n < 1), confirming pseudoplastic (shear-thinning) flow behavior (seen in Table 2). This rheological profile is favorable for electrospinning because it supports polymer chain entanglement and sufficient viscoelasticity at low shear while allowing smooth flow through the needle and stable jet formation under the high-shear conditions of the process. Viscosity is a critical determinant of nanofiber morphology: overly low viscosity can lead to bead formation rather than continuous fibers, whereas excessively high viscosity may cause nozzle clogging and hinder electrospinning [37].

These observations are also consistent with literature evidence. Rošic et al. reported that polymeric solutions showing shear-thinning behavior form more stable jets during electrospinning and yield more uniform fiber diameters [38]. The fact that the solutions producing the most stable jets in the present study also exhibited shear-thinning flow supports this mechanistic interpretation.

A direct comparison between solution P and P(−M) further highlights the impact of micelles on solution rheology. While both systems shared a similar polymeric base, incorporating micelles altered the flow properties: the micelle-loaded solution exhibited a higher yield stress (τ_0_) but lower apparent viscosity, indicating that micelles can modify the internal microstructure of the spinning dope and thereby influence processability. This observation supports the rationale for separately examining the non-micellar counterpart when optimizing composite “micellar nanofiber” inserts.

Based on pre-optimization screening, three representative systems were selected for insert production, i.e., a PVA-dominant system (A), a PVP-dominant system (P), and a blended PVA/PVP system (AP), representing distinct polymer networks and hydration behaviors. In addition to polymer selection, PLU was incorporated in selected compositions as a processing aid to improve electrospinnability and fiber formation by modulating solution properties (e.g., surface tension and viscoelasticity), thereby enhancing jet stability and reducing the likelihood of bead formation. Under the optimized electrospinning parameters, these solutions produced reproducible nanofiber mats suitable for conversion into ocular inserts for subsequent characterization and performance testing.

### 3.3. Characterization of Micellar Nanofiber Inserts

#### 3.3.1. Drug Content and Encapsulation Efficiency

Among the tested formulations, Nanofiber-AP exhibited the highest encapsulation efficiency for both active compounds, whereas the lowest values were obtained for Nanofiber-A. The relatively lower encapsulation efficiency of Nanofiber-A may be attributed to the need for a higher applied voltage during electrospinning (≥25 kV) compared with the other formulations, which could potentially disrupt the micellar structure and compromise drug retention [39]. In addition, the smaller fiber diameter of Nanofiber-A and the thinner insert structure may have contributed to reduced loading; literature reports suggest a positive relationship between insert thickness and encapsulation capacity [40], and Nanofiber-A was thinner than the other formulations (seen in Table 3).

The combined use of PVA and PVP has been reported to enhance drug encapsulation in electrospun systems, likely due to interactions between hydroxyl groups of PVA and carbonyl groups of PVP, which can stabilize the fiber network and increase loading capacity [41,42]. Consistent with this rationale, the higher loading performance of the PVA/PVP blend formulation (Nanofiber-AP) in this study may be associated with improved matrix stability and drug retention. Although the encapsulation efficiencies in nanofiber formulations were generally high, the overall drug loading capacity of inserts remained lower than that of micelles (approximately ~20-fold) because electrospinning requires relatively high polymer content. Nevertheless, the inserts contained PSC at concentrations substantially above its minimum inhibitory concentration (MIC) and DKP at therapeutically relevant levels [43,44]. To confirm content uniformity across the collector-deposited mats, samples were taken from the center and peripheral regions of the nanofiber layer; the encapsulation results were consistent across locations, indicating homogeneous drug distribution.

#### 3.3.2. Thickness, Diameter, and Weight

The maximum thickness of 0.506 ± 0.030 mm for Nanofiber-AP. These thickness values are comparable to commercially available ocular inserts, such as Ocusert^®^ (0.3 mm) and Lacrisert^®^ (1.27 mm), indicating that the produced inserts fall within a practical range for ocular placement. To ensure unit-size uniformity, inserts were prepared using a circular punch (diameter = 5.8 mm). In terms of dimensions, the prepared inserts were compatible with marketed products: Ocusert^®^ is approximately 5.5 mm × 13 mm, and Lacrisert^®^ is approximately 1.27 mm × 3.5 mm [45].

#### 3.3.3. FESEM Imagining and Fiber Diameter

The mean fiber diameters were 185.4 ± 62.5 nm for Nanofiber-A, 229.2 ± 87.7 nm for Nanofiber-P, and 410 ± 139 nm for Nanofiber-AP. The smaller fiber diameter observed for Nanofiber-A may be explained by the higher applied voltage during electrospinning, as increased voltage has been associated with reduced fiber diameter in the literature [46]. Moreover, the relatively lower molecular weight of the PVA used in the present study may also have contributed to smaller fiber diameters [47]. In contrast, Nanofiber-AP exhibited the largest fiber diameter; this trend is consistent with the findings of Rahmani et al., who reported that fibers electrospun from PVA/PVP blends had larger diameters than fibers produced from PVA or PVP alone [48]. Multiple studies have demonstrated hydrogen bonding between the hydroxyl groups of PVA and the carbonyl groups of PVP in blended systems [49,50,51]. Furthermore, partial hydrogen bond formation during post-electrospinning drying has been suggested as water evaporates from the fiber network, potentially promoting fiber–fiber interactions and thickening in PVA/PVP-based mats [52]. These interactions may account for the increased diameter observed for Nanofiber-AP.

#### 3.3.4. Differential Scanning Calorimetry (DSC) Analysis

DSC thermograms showed characteristic endothermic transitions for the pure actives at 101.17–112.35 °C for DKP and 164.26–171.35 °C for PSC, in agreement with previously reported values [53,54]. In the drug-loaded nanofiber formulations, the absence of distinct melting endotherms was interpreted as evidence of a crystalline-to-amorphous transition and/or molecular dispersion of the actives within the polymeric network. Similar loss of melting events has been reported for drug-loaded electrospun systems and has been attributed to amorphization during fiber formation [55].

#### 3.3.5. X-Ray Diffraction (XRD) Mapping

XRD diffractograms (Figure 4) further supported these observations. The diffraction patterns of pure PSC and DKP were consistent with the literature [56,57]. In contrast, nanofiber formulations exhibited a reduction in crystalline features, as reflected by diminished sharp peaks and a more diffuse profile, indicating reduced crystallinity and supporting the formation of an amorphous dispersion in the electrospun matrices. Such peak broadening/attenuation has also been reported for PVP-based drug-loaded nanofibers and is commonly associated with amorphization and loss of long-range order [58].

#### 3.3.6. Fourier-Transform Infrared Spectroscopy (FTIR, ATR-FTIR) Analysis

ATR-FTIR spectroscopy was used to investigate compatibility and intermolecular interactions among the actives and excipients. For DKP, characteristic absorption bands were observed in the 2900–3000 cm^−1^ region (C–H/CH_2_ stretching), at 1658.78 cm^−1^ (C=O stretching), at 1570.06 cm^−1^ (N–H bending), and in the 1300–1400 cm^−1^ region (C–O stretching). For PSC, key bands appeared at 3120.82 cm^−1^ (O–H stretching), 2966.52 cm^−1^ (CH_2_ stretching), 1685.79 cm^−1^ (C=O stretching), 1616.35 cm^−1^ (C=N stretching), 1508.33 cm^−1^ (aromatic skeleton vibrations), and 1450.47 cm^−1^ (C–N stretching). The excipients also showed their expected spectral features: PVA exhibited bands at 3414 cm^−1^ (O–H stretching), 2958.8 cm^−1^ (C–H stretching, CH_2_), 1651.07 cm^−1^ (H–O–H bending of absorbed water), 1423.47 cm^−1^ (C–H bending), bands in the 1049–1226 cm^−1^ region (C–O stretching), and bands in the 840–920 cm^−1^ region (C–C stretching and C–OH bending). PVP showed bands at 3441.01 cm^−1^ (O–H stretching), 2951.09 cm^−1^ (C–H stretching, CH_2_), 1651.07 cm^−1^ (C=O stretching), 1423.42 cm^−1^ (C–H bending), 1284.59 cm^−1^ (C–N stretching), and bands in the 1050–1100 cm^−1^ region (C–O stretching). Pluronic F127 (PLU) displayed prominent bands in the 2916–2850 cm^−1^ region (C–H stretching), in the 1450–1460 cm^−1^ region (C–H bending), at 1249.87 cm^−1^ (in-plane O–H bending), at 1087.85 cm^−1^ (C–O–C stretching of ether bonds), in the 950–960 cm^−1^ region (C–H bending related to alkyl groups), and at 840.96 cm^−1^ (out-of-plane C–H bending, CH_2_). Overall, the spectra of the individual actives and excipients were consistent with literature [59,60,61,62,63].

Notably, while DKP- and PSC-related bands were observable in the physical mixtures, these drug-specific bands were not clearly distinguishable in the nanofiber spectra, which was interpreted as successful incorporation of the actives within the nanofiber system. Considering the dual-carrier design of this work (micelles embedded into electrospun fibers), the ATR-FTIR findings were further interpreted to support effective encapsulation of the drugs in micelles prior to electrospinning and maintenance of the overall carrier structure during fiber formation. In addition, comparison of the spectra of PVA and PVP powders with the PVA/PVP blend nanofiber (Nanofiber-AP) indicated broadening/distortion of O–H stretching bands in the 3000–3600 cm^−1^ region, which was attributed to hydrogen bonding within the blended system [64].

### 3.4. In Vitro Drug Release

When comparing dosage forms, it should be considered that nanofiber inserts inherently contain much lower drug amounts than micellar dispersion because electrospinning requires high polymer content (approximately ~20-fold difference).

DKP exhibited a pronounced burst release across all systems, consistent with its BCS class I character and high aqueous solubility; DKP is also expected to be located mainly in the micellar shell, facilitating rapid release [65]. DKP reached maximum release by 3 h in micelles and by 4 h in most nanofiber formulations, after which a plateau was observed. Among nanofibers, the highest DKP release was obtained with Nanofiber-P(−M) (405.32 ± 10.31 µg/cm^2^; 96.03 ± 2.44%), while the micellar formulation (8999.44 ± 653.51 µg/cm^2^; 79.64 ± 5.78%) and control solution (9552.06 ± 176.00 µg/cm^2^; 84.53 ± 1.55%) produced substantially higher DKP release due to the solution-based presentation (Figure 6).

For PSC, nanofiber formulations generally reached maximum release at 8 h, except Nanofiber-A, which peaked earlier at 6 h, likely due to its smaller fiber diameter that can accelerate release [66]. The micellar formulation reached maximum PSC release at ~3 h, whereas the control solution peaked at 8 h. Among nanofibers, Nanofiber-AP(−M) showed the highest PSC release (5.57 ± 0.39 µg/cm^2^; 65.72 ± 4.68%), followed by Nanofiber-A (5.14 ± 0.21 µg/cm^2^; 60.69 ± 2.54%) and Nanofiber-AP (4.60 ± 0.25 µg/cm^2^; 54.30 ± 3.05%). PSC release from micelles (209.17 ± 13.05 µg/cm^2^; 74.04 ± 4.62%) was markedly higher than from the control solution (44.63 ± 7.76 µg/cm^2^; 15.79 ± 2.74%). A short initial lag was observed for PSC in AP(−M) and P(−M), where release began after 30 min (Figure 7).

One-way ANOVA indicated no significant differences among nanofiber formulations for DKP or PSC release (*p* > 0.05), whereas comparing all systems together (micelles, nanofibers, and control) revealed significant differences for both drugs (*p* < 0.05). Micelles and the control solution differed significantly for PSC release (*p* < 0.05) but not for DKP (*p* > 0.05), likely due to DKP’s rapid release in both cases.

Finally, the PSC release profile of the micellar formulation differed from that reported for PSC-loaded micelles by Durgun et al. [25], with the present study showing a faster PSC release. A plausible explanation is the presence of DKP in the micellar shell: rapid DKP desorption could create transient voids/defects in the micellar structure and thereby facilitate PSC diffusion, accelerating release [65]. This interpretation is supported by literature on combined drug-loaded TPGS micelles; for example, Gaikwad et al. reported that micelles co-loaded with albendazole and paclitaxel exhibited a biphasic release pattern characterized by an initial burst followed by sustained release over time [67].

Release profile fitting using the Korsmeyer–Peppas model supported a predominantly non-Fickian mechanism. For all formulations, the release exponent n values were >0.89 for both DKP and PSC, indicating super case-II transport, which is commonly associated with polymer relaxation/erosion contributions in addition to diffusion.

In such systems, drug release is strongly influenced by physical changes in the polymer matrix, including swelling and erosion, which can lead to a rapid increase in the release rate. This behavior is typically observed when the drug is embedded within a highly swellable polymeric network.

In super case-II transport, drug release does not follow a linear model; instead, it commonly involves an initial burst release followed by a sustained release phase. This release pattern is particularly relevant to the formulation of controlled drug delivery systems, where achieving the desired release profile is critical for therapeutic performance [68]. Consistent with our findings, Rao et al. reported that norfloxacin-loaded ocular inserts prepared using ethyl cellulose and PVP exhibited drug release governed by the super case-II mechanism [69].

### 3.5. Ex Vivo Permeation and Penetration Studies

Noxafil (40 mg/mL) and DKP solution and Nanofiber-AP were evaluated. The reason for eliminating other nanofiber formulations and conducting further studies with Nanofiber-AP is that this formulation exhibits superior overall performance for both APIs, including the highest encapsulation efficiency, clinically acceptable thickness range for ocular implants, and controlled release behavior. In addition, the combined use of PVA and PVP provided improved structural integrity and reproducibility compared to single polymer systems, making Nanofiber-AP the most representative candidate for advanced ex vivo evaluation.

#### 3.5.1. Ex Vivo Permeation Studies

As in the in vitro release study, it should be noted that the drug amount present in the micellar dispersion and the drug amount incorporated into Nanofiber-AP produced from the micellar solution differ markedly due to the high polymer requirement of electrospinning; for the ex vivo studies, this difference was approximately ~20-fold, which should be considered during interpretation.

- Corneal Permeation

For all three systems (micelles, Nanofiber-AP, and control), the highest cumulative permeation of both drugs was observed at 8 h. At 8 h, DKP permeation through the cornea was 39,309.4 ± 3783.19 µg/cm^2^ for the micellar formulation, 26,299.62 ± 2141.29 µg/cm^2^ for the control solution, and 2167.24 ± 559.55 µg/cm^2^ for Nanofiber-AP. At 8 h, PSC permeation through the cornea was 281.56 ± 3.44 µg/cm^2^ for the micellar formulation, 10.90 ± 0.39 µg/cm^2^ for the control solution, and 30.87 ± 0.17 µg/cm^2^ for Nanofiber-AP. For PSC, the first detectable permeation occurred at 1 h in the micellar formulation and Nanofiber-AP, whereas no PSC permeation was detected during the first 6 h for the control solution (Figure 8). The cornea contains both lipophilic and hydrophilic barrier components; the epithelium exhibits lipophilic character, while the stroma is highly hydrophilic and constitutes a major barrier for lipophilic drugs [70,71]. In the control solution, PSC is present as free drug with very low aqueous solubility, and its particle size (1400 nm) exceeds corneal pore dimensions; therefore, only a very low permeation was detected after 6 h [72]. In corneal PSC permeation, the micellar formulation provided the highest permeation, and Nanofiber-AP showed higher permeation than the control solution despite containing approximately 10-fold lower drug concentration. The micellar formulation yielded approximately 28-fold higher PSC permeation than the control solution.

One-way ANOVA results indicated that micelles, Nanofiber-AP, and control solution differed significantly for both DKP and PSC corneal permeation (*p* < 0.05). When micelles were compared with the control solution, PSC corneal permeation differed significantly (*p* < 0.05) while DKP corneal permeation did not (*p* > 0.05). In addition, the difference between Nanofiber-AP and control solutions in corneal PSC permeation was significant (*p* < 0.05), indicating that the micelle-loaded nanofiber system increased corneal PSC permeability compared with the higher-dose control.

- Scleral Permeation

Similar to corneal experiments, the highest permeation values for both drugs were observed at 8 h. At 8 h, DKP permeation through the sclera was 27,137.62 ± 1443.32 µg/cm^2^ for the micellar formulation, 24,815.53 ± 1315.85 µg/cm^2^ for the control solution, and 2077.47 ± 77.03 µg/cm^2^ for Nanofiber-AP. At 8 h, PSC scleral permeation was 334.11 ± 5.36 µg/cm^2^ for the micellar formulation, 155.06 ± 7.47 µg/cm^2^ for the control solution, and 213.39 ± 6.45 µg/cm^2^ for Nanofiber-AP. For the control solution, the first PSC permeation was detected at 1 h (Figure 9). The sclera has a predominantly hydrophilic character and can act as a barrier to lipophilic drugs [73]. One-way ANOVA indicated a significant difference among micelles, Nanofiber-AP, and control solution for scleral permeation (*p* < 0.05). When micelles were compared with the control solution, PSC scleral permeation differed significantly (*p* < 0.05) whereas DKP scleral permeation did not (*p* > 0.05). The difference between Nanofiber-AP and the control solution for PSC scleral permeation was also significant (*p* < 0.05), consistent with the corneal findings.

#### 3.5.2. Ex Vivo Penetration Studies

- Corneal Penetration

For DKP, the highest corneal penetration was observed for the micellar formulation (904.531 ± 187.6 µg), followed by the control solution (725.221 ± 36.45 µg) and Nanofiber-AP (83.723 ± 24.2 µg). For corneal DKP penetration, the three groups differed significantly when evaluated collectively (*p* < 0.05), whereas the comparison between micelles and control solution did not show a significant difference (*p* > 0.05).

For PSC, the highest corneal penetration was obtained with the micellar formulation (5.144 ± 1.581 µg), followed by Nanofiber-AP (3.498 ± 0.177 µg) and the control solution (1.412 ± 0.076 µg). Corneal PSC penetration differed significantly among the three systems (*p* < 0.05). Pairwise comparisons showed significant differences between micelles vs. control solution (*p* < 0.05) and Nanofiber-AP vs. control solution (*p* < 0.05). In contrast, the difference between micelles and Nanofiber-AP for corneal PSC penetration was not significant (*p* > 0.05).

- Scleral Penetration

For DKP, the highest scleral penetration was observed in the control solution (252.999 ± 30.97 µg), followed by the micellar formulation (196.021 ± 43.64 µg) and Nanofiber-AP (34.24 ± 6.578 µg) (Figure 10). Collective analysis showed significant differences among the three groups (*p* < 0.05), while the comparison between micelles and control solution was not significant (*p* > 0.05) for DKP penetration.

For PSC, the highest scleral penetration was obtained with the micellar formulation (10.257 ± 2.654 µg), followed by Nanofiber-AP (3.666 ± 1.266 µg) and the control solution (1.432 ± 0.16 µg) (Figure 10). Scleral PSC penetration differed significantly among all groups (*p* < 0.05). Pairwise comparisons showed significant differences for micelles vs. control solution (*p* < 0.05) and Nanofiber-AP vs. control solution (*p* < 0.05). Unlike the corneal PSC penetration results, the difference between micelles and Nanofiber-AP was significant in the sclera (*p* < 0.05). Overall, PSC penetration was higher in the sclera than in the cornea, consistent with reports indicating that scleral tissue is more permissive to drug penetration compared with the cornea [74,75].

Finally, within-formulation comparisons of penetration between cornea and sclera were performed. In the micellar formulation, both DKP and PSC showed significant differences between corneal and scleral penetration (*p* < 0.05). In Nanofiber-AP, a significant difference was observed for DKP penetration (*p* < 0.05), whereas PSC penetration did not differ significantly between tissues (*p* > 0.05). A similar pattern was obtained for the control solution, with significance for DKP but not for PSC.

The ex vivo findings indicate that PSC transport across ocular tissues is strongly formulation-dependent, whereas DKP transport is comparatively less sensitive to the carrier platform. For permeation, the micellar formulation consistently enabled earlier and higher PSC passage, particularly across the cornea where the control solution showed a pronounced delay, consistent with PSC’s low aqueous solubility and particulate state in the absence of a carrier. Importantly, Nanofiber-AP enhanced PSC permeation and penetration relative to the control solution despite containing ~20-fold lower drug concentration, demonstrating the functional advantage of the micelle-loaded nanofiber architecture for PSC delivery. In contrast, DKP permeation was largely governed by dose and its high intrinsic solubility/permeability (BCS Class I), which also explains why micelles and the control solution did not differ significantly for DKP in pairwise comparisons. Finally, the generally higher PSC deposition in the sclera compared with the cornea aligns with the greater permissiveness of scleral tissue to drug penetration supporting the relevance of scleral delivery routes for lipophilic compounds.

### 3.6. Antifungal Activity (Disk Diffusion)

The results showed that the micellar formulation produced a larger inhibition zone than its concentration-matched control solution (Noxafil^®^ + DKP mixture). Similarly, Nanofiber-AP exhibited a higher inhibition zone diameter compared with its corresponding concentration-matched control. Notably, although Nanofiber-AP contained a lower drug dose than the micellar formulation, it generated an inhibition zone comparable to that of the micelles against *Candida albicans*. This finding was attributed to the fact that Nanofiber-AP still contained PSC at concentrations above the minimum inhibitory concentration (MIC) despite the lower dose [76].

The antifungal activity of drugs or formulations can be demonstrated using many different tests. The disk diffusion test is a standardized qualitative screening method according to CLSI M44-A2 guidelines [77] for comparatively evaluating antifungal activity between formulations containing equivalent or reduced drug concentrations. The primary goal of our study was to determine whether the production of micelles and micellar nanofibers in combination with DKP maintained or enhanced the antifungal effect of PSC. Therefore, minimum inhibitory concentration (MIC) or time-kill assay tests, which would provide a definitive pharmacodynamic result, were not preferred [78]. These studies will be conducted as part of microbiology research that will also be carried out on more advanced clinical strains.

### 3.7. HET-CAM Irritation Test

The observed slight irritation response associated with the diluted Noxafil^®^ suspension may be attributed to the presence of formulation excipients and the relatively high local drug concentration upon direct CAM exposure. Importantly, the absence of coagulation, hemorrhage, or vascular lysis for all micelle-loaded nanofiber formulations indicates favorable ocular tolerability of the developed delivery systems. These findings suggest that incorporation of the active compounds into polymeric micelles and electrospun nanofibers effectively mitigates irritation potential, supporting the suitability of the formulations for ocular application. The obtained results are consistent with previously reported HET-CAM findings for similar nanocarrier-based ocular systems, including those reported by Durgun et al. [79].

## 4. Materials and Methods

### 4.1. Materials

Posaconazole (PSC) was obtained from MSN Laboratories (Hyderabad, India). Dexketoprofen trometamol (DKP) was kindly provided as a gift sample by Santa Farma (İstanbul, Türkiye). D-α-tocopheryl polyethylene glycol 1000 succinate (TPGS), poly(vinyl alcohol) (PVA; Mw: 50 kDa), poly(vinylpyrrolidone) (PVP; Mw: 1200 kDa), and Pluronic^®^ F127 (PLU) were obtained from BASF Pharma (Ludwigshafen, Germany). Methanol (high-performance liquid chromatography (HPLC) grade), acetonitrile (HPLC grade), sodium lauryl sulfate (SLS) and sodium chloride (NaCl) were purchased from Merck (Darmstadt, Germany) and used as received. Isotonic NaCl solution (0.9% *w*/*v*) was purchased from Polifarma (Tekirdağ, Türkiye). Ultrapure water was obtained using a Milli-Q purification system (Millipore, MA, USA). All remaining reagents were of analytical grade.

Electrospinning was performed using an NE-200 electrospinning system (Inovenso, Boston, MA, USA). Dynamic light scattering (DLS) and zeta potential measurements were performed using a Zetasizer Nano ZS (Malvern Panalytical, Malvern, UK). HPLC analyses were conducted using a Shimadzu HPLC system (Shimadzu, Kyoto, Japan). ATR-FTIR spectra and DSC analyses were obtained using Shimadzu instruments (Shimadzu, Kyoto, Japan). Rheological measurements were performed using a Haake RheoStress 1 rheometer (Haake, Karlsruhe, Germany). Conductivity was measured using an Eutech Instruments PC2700 conductivity meter (Eutech Instruments, Landsmeer, The Netherlands). FESEM imaging was performed using a Thermo Scientific Apreo 2S FESEM (Thermo Fisher Scientific, Waltham, MA, USA).

### 4.2. Analytical Method

Quantification of PSC and DKP was carried out by HPLC using a C18 column (Inertsil ODS-3, 5 µm, 4.6 × 250 mm). The mobile phase consisted of methanol:water (80:20, *v*/*v*) adjusted to pH 3.5 with HCl. The flow rate was 0.8 mL/min, the column temperature was maintained at 35 °C, the injection volume was 20 µL, and UV detection was set at 262 nm.

Validation studies were performed in accordance with the ICH Q2(R1) guideline, assessing linearity, accuracy, precision, selectivity, and sensitivity (LOD/LOQ) [80].

### 4.3. Preparation and Characterization of PSC/DKP-Loaded Polymeric Micelles

#### 4.3.1. Preparation of PSC/DKP-Loaded Polymeric Micelles

PSC/DKP-loaded polymeric micelles were prepared by a thin-film hydration method modified from the procedure reported by Durgun et al. [25]. Briefly, TPGS was dissolved in acetonitrile. PSC and DKP were dissolved separately in methanol and then added dropwise to the TPGS solution under magnetic stirring. The resulting mixture was transferred to a round-bottom flask and evaporated using a rotary evaporator (IKA^®^-Werke GmbH & Co. KG, Staufen, Germany) at 60 °C (120 rpm, 20 min). The pressure was then reduced to 150 mbar to remove residual solvents, forming a thin film. After an additional resting period (20 min), the film was hydrated with isotonic saline (0.9% NaCl) and mixed on the rotary evaporator at 60 °C (120 rpm) for 1 h (without changing ambient pressure). The dispersion was allowed to stand for 1 h and then filtered through a 0.45 µm PTFE membrane.

The optimized micelle formulation (M) contained PSC (250 µg/mL), DKP (10 mg/mL), and TPGS (20 mg/mL) (Table 7).

#### 4.3.2. Characterization of PSC/DKP-Loaded Polymeric Micelles

- Particle Size, Polydispersity Index (PDI), and Size Distribution

The hydrodynamic diameter and polydispersity index (PDI) of the micellar dispersions were determined by dynamic light scattering (DLS) at 25 °C using a Zetasizer Nano ZS (Malvern Panalytical, UK). Micellar dispersions were gently homogenized and transferred into disposable polystyrene cuvettes prior to measurement. Each sample was analyzed in triplicate, and each measurement consisted of 10 consecutive runs. Results were reported as mean ± standard deviation together with the intensity-based size distribution.

- Zeta Potential

Zeta potential was measured by electrophoretic light scattering using the same instrument at 25 °C. Samples were loaded into folded capillary cells according to the manufacturer’s instructions, and zeta potential values were calculated using the Smoluchowski approximation [81]. Measurements were conducted in triplicate (10 runs per measurement), and results were expressed as mean ± standard deviation.

- Determination of Drug Content and Encapsulation efficiency (*EE%*)

To quantify PSC and DKP incorporated into the micellar system, micelles were disrupted with methanol to ensure complete extraction of both drugs. Briefly, 0.1 mL of micellar dispersion was diluted to 1.0 mL with methanol (1:10, *v*/*v*) and mixed thoroughly until a clear solution was obtained. The resulting solutions were analyzed by HPLC. Drug concentrations were calculated using the corresponding calibration curves.

Encapsulation efficiency (*EE%*) was calculated as the percentage of drug incorporated into the micellar formulation relative to the total amount of drug initially used in the preparation, using the following equation:

(1) $$EE\left(\%\right)=\frac{W_{enc}}{W_{total}}\times 100$$

where $W_{enc}$ is the amount of PSC or DKP quantified after micelle disruption and $W_{total}$ is the total amount of the respective drug (PSC or DKP) used during micelle preparation.

### 4.4. Optimization and Preparation of Micellar Electrospun Nanofiber Ocular Inserts

Polymeric spinning solutions were prepared using poly(vinyl alcohol) (PVA) and poly(vinylpyrrolidone) (PVP) as the main matrix-forming polymers. For blank nanofiber screening, PVA aqueous solutions were prepared in the range of 15–30% (*w*/*w*) by gradually dispersing PVA in distilled water, followed by continuous stirring at 55 °C (350 rpm, 5 h) until a clear and homogeneous solution was obtained. PVP aqueous solutions were prepared in the range of 8–25% (*w*/*w*) by dissolving PVP in distilled water under magnetic stirring at room temperature (400 rpm, 5 h) until complete dissolution. For PVA/PVP blend nanofibers, polymer blends were screened at a fixed total polymer content of 24% (*w*/*w*), while varying the PVA:PVP ratio from 20:4 to 4:20 (*w*/*w*) (i.e., 20/4, 18/6, 16/8, 12/12, 8/16, 6/18, and 4/20). Blended solutions were prepared by mixing the corresponding pre-prepared PVA and PVP solutions and further stirring at 300 rpm for 3 h to ensure uniformity.

Electrospun nanofibers were produced using an electrospinning system (Inovenso, USA). During process development, key electrospinning parameters (nozzle/needle type, applied voltage, flow rate, and tip-to-collector distance) were screened to obtain continuous, bead-free fibers and stable jet formation. Nanofibers were collected on an aluminum foil-covered collector and removed as nonwoven mats.

Following blank (drug-free) optimization, micelle-loaded nanofiber inserts were prepared by using the optimized PSC/DKP-loaded micellar dispersion as the solvent phase. In brief, the required amounts of PVA and/or PVP (and Pluronic F127, when applicable) were dissolved directly in the micellar dispersion under stirring until homogeneous spinning solutions were obtained. In micelle-based systems, preliminary trials were performed using single-polymer systems (PVA: 20–30% (*w*/*w*); PVP: 15–25% (*w*/*w*)) and PVA/PVP blends (total polymer 24% (*w*/*w*), PVA:PVP 20:4 to 4:20). In addition, Pluronic F127 (PLU) was incorporated into selected formulations as a processing aid to improve electrospinnability and fiber formation. Specifically, PLU was used to enhance jet stability and facilitate continuous, bead-free fiber production by modulating the physicochemical properties of the spinning solution (e.g., viscoelasticity and surface tension), thereby improving the overall uniformity of the electrospun mats.

**Figure 12:**
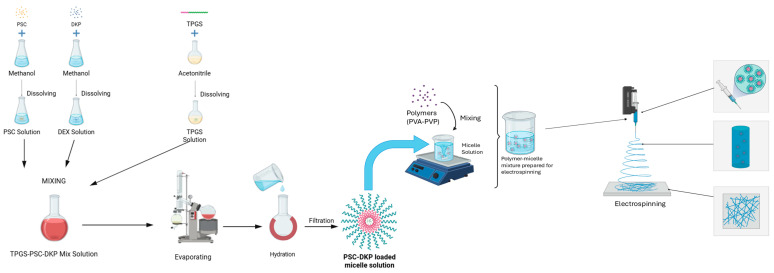
Schematic representation of the preparation of the micelle solution and micelle-loaded nanofibers.

Based on the preformulation/optimization outcomes, three micelle-loaded nanofiber formulations—one PVA-based, one PVP-based, and one PVA/PVP blend—were selected for further studies. The optimized compositions and electrospinning conditions are summarized in Table 8. Unless otherwise stated, electrospinning was carried out using a 0.8 mm nozzle, a flow rate of 0.3 mL/h, and a tip-to-collector distance of 18 cm, while the applied voltage was adjusted according to the formulation (Table 8). After fabrication, nanofiber mats were cut into insert units of predefined dimensions and stored in sealed packaging until characterization and performance testing. The preparation process for micelles and micelle-loaded nanofibers is schematically illustrated in Figure 12.

For comparative assessment of the role of micellar encapsulation, control nanofibers were prepared by incorporating PSC and DKP directly into the corresponding polymer solutions (without micelles) and electrospinning under comparable conditions, as defined in the study design.

### 4.5. Characterization of Spinning Solutions

#### 4.5.1. Rheology

Polymer–micelle spinning solutions were analyzed at 25 °C using a cone-plate geometry (C35 TiL; 53 µm gap; 1° cone angle). Measurements were performed over a shear rate range of 0.5–1000 s^−1^ for 60 s with 100 data points and repeated three times. Data was processed using RheoWin3 software and fitted using the power-law model.

#### 4.5.2. Electrical Conductivity

Spinning solutions (20 g) were prepared in beakers and conductivity was measured at room temperature using a PC2700 conductivity meter.

### 4.6. Characterization of Nanofiber Inserts

#### 4.6.1. Drug Content and Encapsulation Efficiency

Nanofibers (10 mg) were dissolved in 2 mL of distilled water:methanol (2:8, *v*/*v*) and shaken at 300 rpm for 24 h. Samples were centrifuged at 15,000 rpm, filtered through a 0.45 µm PTFE membrane, and analyzed by HPLC (*n* = 3).

#### 4.6.2. Thickness, Diameter, and Weight

Inserts were punched into defined shapes; thickness/diameter were measured with an electronic caliper and weight with an analytical balance (*n* = 3).

#### 4.6.3. FESEM Imaging and Fiber Diameter

Samples were sputter-coated with a thin gold layer prior to FESEM imaging. Fiber diameters were quantified using ImageJ software (version 1.54) from FESEM micrographs (50 measurements per formulation), and diameter histograms were plotted.

#### 4.6.4. Differential Scanning Calorimetry (DSC)

DSC analyses were performed to determine the heat absorbed or released by micelle-loaded nanofibers during heating/cooling or isothermal conditions. Three different nanofiber formulations were evaluated. Samples were analyzed individually under a nitrogen atmosphere at a heating rate of 10 °C/min over 25–300 °C using aluminum pans. Thermograms of the nanofibers, active ingredients, and excipients were compared to assess potential incompatibilities.

#### 4.6.5. X-Ray Diffraction (XRD)

Micelle-loaded nanofibers, active ingredients, and excipients were analyzed using a wide-angle X-ray diffractometer with a CuKα radiation source (λ = 0.154 nm) operated at 40 kV and 30 mA. Diffraction patterns were recorded over a 2θ range of 2° to 40° with a step size of 0.02°. This analysis was conducted to evaluate the crystallographic properties of the nanofiber formulations and identify differences in their solid-state characteristics.

#### 4.6.6. Fourier-Transform Infrared Spectroscopy (FTIR, ATR-FTIR)

ATR-FTIR spectra of the active ingredients, polymers, their physical mixtures, and the produced nanofibers were collected using an ATR-FTIR instrument (Shimadzu, Kyoto, Japan). After placing the nanofibers on the ATR crystal, scans were recorded in the 4000–650 cm^−1^ range at a resolution of 4 cm^−1^. This analysis aimed to investigate possible interactions between active compounds and excipients within the formulations.

### 4.7. In Vitro Release Studies

A receptor medium was selected to ensure sink conditions and reliable quantification of PSC and DKP during in vitro release testing. Based on preliminary screening, artificial tear fluid (ATF, pH 7.4) supplemented with 0.1% (*w*/*v*) sodium lauryl sulfate (SLS) was selected as the release/receptor medium, as it provided sufficient solubilization capacity for both drugs and enabled reproducible sampling throughout the experiments. ATF was prepared by dissolving NaCl (0.67 g), NaHCO_3_ (0.20 g), CaCl_2_·2H_2_O (0.008 g), and KCl (0.14 g) in 100 mL ultrapure water and adjusting the pH to 7.4 with HCl.

In vitro release studies were performed using vertical Franz diffusion cells (receptor volume: 12 mL) equipped with a cellulose acetate membrane. The receptor compartment was filled with ATF containing 0.1% (*w*/*v*) SLS and equilibrated at 35.5 °C under continuous stirring to mimic the ocular surface temperature and maintain homogeneous mixing. For micelle formulations, 2 mL of PSC/DKP-loaded micellar dispersion was placed in the donor compartment. For comparator testing, diluted Noxafil^®^ oral suspension and an aqueous DKP solution were prepared at concentrations matching those of the micellar formulation and tested under the same conditions. For nanofiber inserts, selected electrospun formulations were placed in the donor compartment; to simulate ocular hydration, 1 mL of the receptor medium was added onto the donor side to wet the insert. Control nanofibers prepared without micelles (drug directly incorporated into polymer solutions) were also evaluated in parallel.

At predetermined time points (30, 60, 120, 180, 240, 360, and 480 min), 1 mL samples were withdrawn from the receptor compartment and immediately replaced with an equal volume of fresh, pre-warmed medium to maintain constant volume and sink conditions. Samples were quantified by HPLC. Cumulative drug release was calculated and expressed as released amount per diffusion area (µg/cm^2^) and/or percentage release, as appropriate (*n* = 3).

Release profiles of PSC and DKP from micelles and nanofibers were evaluated using KinetDS 3.0 software by fitting to appropriate kinetic models.

### 4.8. Ex Vivo Permeation and Penetration Studies

#### 4.8.1. Tissue Preparation

Isolated bovine eyes were used for ex vivo studies. Until experimentation, eyes were stored at +4 °C in 0.9% isotonic NaCl solution containing penicillin G (50 U/mL) and streptomycin (50 U/mL), in accordance with the relevant guidelines. Prior to experiments, the eyes were rinsed with 0.9% isotonic NaCl to remove antibiotics and were subsequently kept for 24 h at +4 °C in the same solution. Cornea and sclera tissues were then carefully excised for ex vivo permeation experiments.

#### 4.8.2. Permeation of PSC and DKP Through Ocular Tissues

Ex vivo permeation experiments were conducted using Valia–Chien diffusion cells. The donor and receptor chamber volumes were 2.0 mL for cornea and 3.4 mL for sclera, with diffusion areas of 20.7 mm^2^ (cornea) and 7.2 mm^2^ (sclera).

The receptor medium was artificial tear fluid containing 0.1% (*w*/*v*) SLS, and it was allowed to equilibrate to 35.5 °C for 30 min prior to starting the experiment. Subsequently, receptor medium and test formulations were added (2.0 mL for cornea and 3.4 mL for sclera) ensuring that no air bubbles remained; the excised tissue was positioned between donor and receptor compartments.

Permeation was evaluated comparatively for the micelle formulation, a control mixture consisting of Noxafil^®^ diluted to 250 µg/mL PSC + DKP solution 10 mg/mL, and the selected nanofiber insert formulation (Nanofiber-AP) (*n* = 3). For the nanofiber condition, the insert was placed into the donor compartment and then completed with the receptor medium. Experiments were performed under stirring at 400 rpm. Samples (0.5 mL) were withdrawn from the receptor compartment at 0.5, 1, 2, 3, 4, 6, and 8 h, and the sampled volume was immediately replaced with an equal volume of fresh receptor medium. Collected samples were analyzed by HPLC after appropriate dilution. Cumulative permeated amounts over 8 h were calculated.

#### 4.8.3. Permeation Parameters and Equations

To determine permeation parameters for both PSC and DKP across cornea and sclera, the apparent permeability coefficient (*P_app_*), effective diffusion coefficient (*D_eff_*), and steady-state flux (*J_ss_*) were calculated using the equations provided:

(2) $$P_{app}=\frac{Q}{A\cdot C_{0}}$$

(3) $$D_{eff}=P_{app}\cdot l$$

(4) $$J_{ss}=\frac{\Delta Q}{\Delta t}$$

where Q is the total amount of drug permeated at time t, $C_{0}$ is the initial drug concentration in the donor phase, A is the tissue surface area, and l is the tissue thickness.

#### 4.8.4. Penetration of PSC and DKP Across Ocular Tissues

To quantify tissue penetration, cornea and sclera were collected immediately after completion of the 8 h permeation study. Tissue integrity was confirmed by visual inspection. Each tissue sample was immersed in 4 mL methanol and extracted for 24 h on an orbital shaker. After extraction, the tissue–solvent mixture was transferred into a capped centrifuge tube and centrifuged for 10 min. The supernatant was then filtered through a 0.45 µm PTFE membrane filter, and following necessary dilutions, the amount of PSC and DKP penetrated into ocular tissues was quantified by HPLC.

### 4.9. Antifungal Activity (Disk Diffusion)

A disk diffusion assay was performed to evaluate the antifungal activity of the prepared formulations. *Candida albicans* (ATCC 90028) was used as the test organism. The assay was conducted according to the Clinical and Laboratory Standards Institute (CLSI) M44-A2 guideline [77]. Briefly, fungal suspensions (10^6^ CFU/mL) were spread onto Mueller–Hinton agar plates (Merck, Darmstadt, Germany) (10 cm diameter) containing 25 mL medium supplemented with 2% dextrose and 0.5 mg/L methylene blue. After drying for 20 min, sterile paper disks (6 mm diameter) were placed on the agar surface. Each disk was loaded with 10 µL of the following samples: placebo, micellar formulation, the nanofiber formulation dissolved in water (Nanofiber-AP), a Noxafil^®^/DKP mixture containing active ingredients at concentrations matching the micellar formulation, and a Noxafil^®^/DKP mixture containing active ingredients at concentrations matching the nanofiber formulation. Plates were incubated at 37 ± 0.5 °C for 48 h, and inhibition zone diameters were measured in millimeters.

### 4.10. HET-CAM Irritation Test

The Hen’s Egg Test–Chorioallantoic Membrane (HET-CAM) assay was used to assess the ocular irritation potential of the test samples as an alternative to the Draize rabbit eye test [82]. All experiments were conducted in accordance with ICCVAM recommendations [83]. Fresh, fertilized White Leghorn chicken eggs (50–60 g; not older than 7 days) were used, while excessively deformed eggs and those with cracks or thin shells were excluded. Eggs were incubated for 9 days in an automatically rotating incubator at 37.8 ± 0.3 °C and 58 ± 2% relative humidity. On day 9, eggs were candled to remove non-viable or defective specimens, and the air-cell region was marked. The marked area was opened using a rotary dental saw to access the air chamber, and the shell was carefully removed without damaging the inner membrane to expose the CAM.

Diluted Noxafil^®^ oral suspension/DKP solution mixture, PSC-DKP loaded micelles, nanofiber (Nanofiber-AP) formulations, and their corresponding placebos were tested. A total volume of 0.3 mL of each liquid sample was applied directly onto the vascular CAM. For nanofiber formulations, the insert was placed on the CAM and then wetted with 0.3 mL isotonic water. 0.1 N NaOH and 0.9% (*w*/*v*) NaCl served as positive and negative controls, respectively. CAM responses were observed for 300 s, and the onset times of the predefined endpoints (hemorrhage, lysis, and coagulation) were recorded. Irritation scores were assigned based on the time-to-endpoint scoring scheme (Table 9). Each egg/sample was scored individually; mean scores were calculated for each test group and used to classify the irritation potential (Table 10).

### 4.11. Statistical Analysis

Statistical analysis was performed using GraphPad Prism 8.1.1. One-way ANOVA was applied for comparisons involving more than two groups, while Student’s *t*-test was used for two-group comparisons.

## 5. Conclusions

In this study, innovative “micellar nanofiber” systems were developed for the treatment of ocular fungal infections by combining the broad-spectrum antifungal posaconazole (PSC) with the potent anti-inflammatory agent dexketoprofen trometamol (DKP) in both liquid (micellar) and solid (nanofiber) dosage forms. This research is distinctive in that it represents the first study to investigate ocular application of DKP and brings both agents together within a single dosage form. Within this scope, the effects of using PVA and PVP polymers, as well as their combinations, on formulation performance were also investigated. The prepared micelles exhibited an average size of 11.01 nm, while the electrospun nanofibers showed diameters within the 185–410 nm range, indicating suitable dimensions for ocular delivery. Thermal and structural analyses demonstrated that the crystalline drugs were encapsulated within the nanofiber matrix in an amorphous state, thereby significantly improving the aqueous solubility of the lipophilic PSC. According to the Korsmeyer–Peppas model, these systems provided controlled release via a super case-II transport mechanism and, in ex vivo studies, exhibited markedly higher tissue penetration and permeability in bovine cornea and sclera compared with diluted Noxafil^®^ solution, even at lower doses for the nanofiber inserts. In addition, the developed formulations showed strong antifungal activity against *Candida albicans* and were found to be non-irritant to ocular tissues based on the HET-CAM assay. Overall, these findings indicate that the combined nanocarrier systems may offer an innovative therapeutic alternative for resistant ocular infections by simultaneously targeting infection and inflammation while improving bioavailability. In vivo studies were intentionally not included at this stage, in accordance with the 3R principles (Replacement, Reduction, and Refinement) in animal experimentation, to avoid unnecessary animal use prior to the identification of an optimized formulation. The results obtained provide a robust foundation for future investigations.

Based on these findings, further studies may focus on optimizing the formulation using different polymers or surfactants, as well as advanced techniques such as coaxial electrospinning to enhance drug loading capacity and prolong therapeutic effects. Subsequently, in vivo studies will be conducted to evaluate the therapeutic efficacy and safety of the optimized formulations.

## Figures and Tables

**Table 1:** Physicochemical characteristics of the optimized PSC/DKP-loaded micellar formulation (*n* = 3).

Attribute	Value
Particle size (nm)	11.01 ± 0.06
PDI	0.078
Zeta potential (mV)	−1.68 ± 0.23
Encapsulation efficiency DKP (%)	97.55 ± 4.10
Encapsulation efficiency PSC (%)	98.67 ± 4.71

**Table 2:** Rheological data (Herschel–Bulkley) and electrical conductivity of electrospinning solutions (*n* = 3) (Mean ± SD).

Solution	τ_0_	K	n	r	Conductivity (µS/cm)
A	−43.21 ± 8.76	10.96 ± 1.09	0.68 ± 0.02	0.9997	5732 ± 6.24
P	−17.67 ± 2.78	6.76 ± 0.52	0.65 ± 0.01	0.9999	7490.67 ± 22.28
AP	−123.17 ± 57.33	35.89 ± 16.15	0.57 ± 0.07	0.9995	5920.33 ± 9.29
P(−M)	−50.80 ± 28.48	10.81 ± 6.27	0.64 ± 0.08	0.9996	2139 ± 6.24

τ_0_: The yield stress, K: The consistency index (Pa·s), n: The flow behavior index, r: The correlation coefficient.

**Table 3:** Nanofiber insert properties and drug content (*n* = 3) (Mean ± SD).

Nanofiber	Fiber Diameter (nm)	Insert Diameter (mm)	Thickness (mm)	Weight (mg)	DKP (*EE%*)	PSC (*EE%*)
NF-A	185.4 ± 62.5	5.80 ± 0.01	0.32 ± 0.01	3.62 ± 0.08	86.65 ± 2.75	75.48 ± 5.30
NF-P	229.2 ± 87.7	5.80 ± 0.03	0.44 ± 0.03	5.42 ± 0.06	93.47 ± 1.59	90.33 ± 3.42
NF-AP	410 ± 139	5.80 ± 0.01	0.51 ± 0.03	8.32 ± 0.16	94.78 ± 1.18	91.51 ± 5.37

**Table 4:** Corneal and scleral permeation parameters of Micelle, Nanofiber-AP and or F68 and control (Noxafil^®^ + DKP solution) (*n* = 3) (Mean ± SD).

			*P_app_* (cm s^−1^)		*D_eff_* (s^−1^)		*J_ss_* (µg min^−1^)	
			Mean	±SD	Mean	±SD	Mean	±SD
Cornea	DKP	Micelle	5.69 × 10^−5^	6.59 × 10^−5^	2.22 × 10^−5^	8.38 × 10^−6^	2.31 × 10^−9^	3.41 × 10^−10^
		Nanofiber-AP	2.84 × 10^−5^	2.99 × 10^−5^	1.25 × 10^−5^	6.10 × 10^−6^	1.28 × 10^−10^	3.30 × 10^−11^
		Control	4.15 × 10^−5^	5.25 × 10^−5^	1.45 × 10^−5^	3.96 × 10^−6^	1.55 × 10^−9^	1.85 × 10^−10^
Sclera	DKP	Micelle	1.37 × 10^−5^	1.63 × 10^−5^	4.84 × 10^−6^	2.42 × 10^−6^	5.56 × 10^−10^	2.96 × 10^−11^
		Nanofiber-AP	5.63 × 10^−6^	1.79 × 10^−5^	4.39 × 10^−6^	2.35 × 10^−6^	4.26 × 10^−11^	1.58 × 10^−12^
		Control	6.93 × 10^−6^	2.15 × 10^−5^	5.22 × 10^−6^	2.74 × 10^−6^	5.08 × 10^−10^	2.70 × 10^−11^
Cornea	PSC	Micelle	4.58 × 10^−8^	6.99 × 10^−8^	9.38 × 10^−9^	2.88 × 10^−9^	6.02 × 10^−15^	7.36 × 10^−16^
		Nanofiber-AP	4.28 × 10^−8^	6.29 × 10^−8^	1.01 × 10^−8^	1.28 × 10^−9^	6.60 × 10^−16^	3.73 × 10^−17^
		Control	1.65 × 10^−9^	2.48 × 10^−9^	3.60 × 10^−10^	7.95 × 10^−11^	2.33 × 10^−16^	8.38 × 10^−18^
Sclera	PSC	Micelle	1.93 × 10^−8^	2.99 × 10^−8^	3.17 × 10^−9^	3.87 × 10^−10^	2.45 × 10^−15^	4.14 × 10^−16^
		Nanofiber-AP	6.07 × 10^−8^	8.63 × 10^−8^	1.51 × 10^−8^	7.77 × 10^−9^	1.15 × 10^−15^	5.56 × 10^−16^
		Control	8.40 × 10^−9^	1.20 × 10^−8^	2.01 × 10^−9^	8.37 × 10^−10^	1.54 × 10^−15^	5.88 × 10^−16^

To calculate corneal *D_eff_*, the thickness of bovine cornea and sclera was assumed as 0.1 cm and 0.065 cm, respectively.

**Table 5:** Zone diameters (mm) determined with the disk diffusion method of the Placebos, Micelles, Nanofiber-AP, and Controls (*n* = 4) (against *Candida albicans*).

Formulation	Inhibition Zone (mm) (Mean ± SD)
Placebo for micelle solution	0
Placebo for Nanofiber-AP	0
Micelles	26.25 ± 0.50
Nanofiber-AP	24.25 ± 0.50
Control mixture (same concentration as Micelle solution)	24.50 ± 0.58
Control mixture (same concentration as Nanofiber-AP)	22.00 ± 0.41

**Table 6:** Cumulative scores and HET-CAM irritation assessment of Micelle, Nanofiber-AP, Placebos and Controls (*n* = 3).

Formulation	Cumulative Score	Irritation Assessment
Placebo (Micelle)	0	Practically none
Placebo (Nanofiber-AP)	0	Practically none
Micelle solution	0	Practically none
Nanofiber-AP	0	Practically none
DKP solution	0	Practically none
Diluted Noxafil^®^	3	Slight
Mixture of Noxafil^®^-DKP	0	Practically none

**Table 7:** Optimized formulation of PSC and DKP loaded micellar system.

Micelle Formulation Code	PSC Concentration	DKP Concentration	TPGS Concentration
M	250 µg/mL	10 mg/mL	20 mg/mL

**Table 8:** Optimized micelle-loaded nanofiber formulations and electrospinning parameters (solvent phase: micellar dispersion).

Nanofiber Formulation Code	Composition (*w*/*w*, %)	Flow Rate (mL/h)	Distance (cm)	Voltage (kV)	Nozzle
Nanofiber-AP (NF-AP)	PVA (12) + PVP (12)	0.3	18	20	0.8 mm
Nanofiber-A (NF-A)	PVA (24) + PLU (1)	0.3	18	25	0.8 mm
Nanofiber-P (NF-P)	PVP (17) + PLU (1)	0.3	18	20	0.8 mm

**Table 9:** Scoring scheme for the HET-CAM test for membrane irritation.

Effect	Score at 30 s	Score at 120 s	Score at 300 s
Lysis	5	3	1
Hemorrhage	7	5	3
Coagulation	9	7	5

**Table 10:** Classification scheme for cumulative scores in the HET-CAM test.

Cumulative Score	Irritation Assessment
Up to 0.9	Practically none
1—4.9	Slight
5—8.9	Moderate
9 and above	Strong

## Data Availability

This article is based on data obtained from Egemen Uzel’s doctoral thesis. Data sharing is confidential as the study is currently undergoing a patent application process.

## Abbreviations

The following abbreviations are used in this manuscript:

CMC	Critic Micelle Concentration
COX	Cyclooxygenase
DKP	Dexketoprofen trometamol
DLS	Dynamic light scattering
DSC	Differential Scanning Calorimetry
FTIR	Fourier-Transform Infrared
HET-CAM	The Hen’s Egg Test–Chorioallantoic Membrane
HPLC	High-performance liquid chromatography
NaCl	Sodium chloride
NF	Nanofiber
PDI	Polydispersity Index
PLU	Pluronic F127
PSC	Posaconazole
PVA	poly(vinyl alcohol)
PVP	poly(vinylpyrrolidone)
SLS	Sodium lauryl sulfate
TPGS	D-α-tocopheryl polyethylene glycol 1000 succinate
